# Combining Next Generation Sequencing with Bulked Segregant Analysis to Fine Map a Stem Moisture Locus in Sorghum (*Sorghum bicolor* L. Moench)

**DOI:** 10.1371/journal.pone.0127065

**Published:** 2015-05-18

**Authors:** Yucui Han, Peng Lv, Shenglin Hou, Suying Li, Guisu Ji, Xue Ma, Ruiheng Du, Guoqing Liu

**Affiliations:** 1 Key Laboratory of Minor Cereal Crops in Hebei Province/Department of Biotechnology, Institute of Millet Crops, Hebei Academy of Agricultural & Forestry Sciences, Shijiazhuang, China; 2 Hebei Branch of the National Sorghum Improvement Center/ Department of Sorghum Breeding, Institute of Millet Crops, Hebei Academy of Agricultural & Forestry Sciences, Shijiazhuang, China; College of Agricultural Sciences, UNITED STATES

## Abstract

Sorghum is one of the most promising bioenergy crops. Stem juice yield, together with stem sugar concentration, determines sugar yield in sweet sorghum. Bulked segregant analysis (BSA) is a gene mapping technique for identifying genomic regions containing genetic loci affecting a trait of interest that when combined with deep sequencing could effectively accelerate the gene mapping process. In this study, a dry stem sorghum landrace was characterized and the stem water controlling locus, *qSW6*, was fine mapped using QTL analysis and the combined BSA and deep sequencing technologies. Results showed that: (i) In sorghum variety Jiliang 2, stem water content was around 80% before flowering stage. It dropped to 75% during grain filling with little difference between different internodes. In landrace G21, stem water content keeps dropping after the flag leaf stage. The drop from 71% at flowering time progressed to 60% at grain filling time. Large differences exist between different internodes with the lowest (51%) at the 7^th^ and 8^th^ internodes at dough stage. (ii) A quantitative trait locus (QTL) controlling stem water content mapped on chromosome 6 between SSR markers Ch6-2 and gpsb069 explained about 34.7-56.9% of the phenotypic variation for the 5^th^ to 10^th^ internodes, respectively. (iii) BSA and deep sequencing analysis narrowed the associated region to 339 kb containing 38 putative genes. The results could help reveal molecular mechanisms underlying juice yield of sorghum and thus to improve total sugar yield.

## Introduction

Sorghum (*Sorghum bicolor* L. Moench) is one of the most important bioenergy crops in the grass family (Poaceae) that employs C_4_ photosynthesis and is capable of producing high biomass yield in the form of lignocellulose, fermentable juice, and fermentable grain [1]. It is highly resistant to abiotic factors such as drought, salinity and soil alkalinity. The soluble sugars (sucrose, glucose and fructose) accumulated in its stalks can reach up to 19% of the stem fresh weight [2] and can be directly fermented into ethanol or other forms of biological fuel with conversion efficiencies of more than 90% [3]. Up to 13.2 t/ha of total sugars, equivalent to 7,682 L of ethanol per hectare can be produced by sweet sorghum under favorable conditions [4]. Furthermore, after juice extraction, the crushed residue or bagasse can be processed as lignocellulosic biomass which could also be converted to ethanol or used for other traditional applications. Thus sorghum has become a model system for bioenergy crops.

Total sugar yield is determined by juice yield and soluble solid content [5]. Previous studies focused on soluble solid content (brix) and quite a few quantitative loci controlling the trait have been mapped on chromosomes 1, 2, 3, 4, 5 and 7 using both family-based linkage analysis and natural population-based association analysis [1,6–16]. Mapping results from these studies vary but are consistent with the conclusion that most QTLs act in an additive manner, which means that both hybrid parents should have high brix values in order to obtain high brix offspring. In addition, percent soluble solids has a physiological limit of approximately 25%, thus, like in sugarcane, focusing on increased juice yield is the most efficient method of increasing total sugar production [17].

Previous studies have indicated that juice yield is affected by many genes (i.e. is subject to polygenic control), and by gene–environment interactions. Some quantitative trait loci (QTLs) affecting juice yield have been isolated in different sorghum mapping populations and found to be located on chromosomes 1, 4, 6 and 9 with the phenotype variation explanation of 7.7–77.0% [7,8,18]. However, one common drawback in the previous studies was little percent moisture existed between the parental strains for mapping population construction, which, although transgressive segregations in intercross populations are often observed, could reduce the power and accuracy to detect QTL[19–21]. So an ideal mapping population is usually developed by crossing two inbred parents with clear contrasting difference in phenotypic trait(s) of interest. Thus in order to accurately map the loci (genes) and better understand the molecular mechanisms underlying percent moisture, we developed a new mapping population using parental materials with highly significant difference in percent moisture.

Bulked-segregant analysis (BSA) is traditionally used to identify DNA markers tightly linked to target gene (s) for a given phenotype. It has been widely applied for gene mapping but it requires DNA marker development and genotyping which is time consuming and labor intensive. Next generation sequencing (NGS) technologies are providing new ways to accelerate fine-mapping and gene isolation [22,23]. Combining the two technologies has proven to be successful for efficient gene mapping in plants [24].

In the present study, the dry stem trait in a sorghum landrace, G21, was characterized. QTL analysis was conducted using an F_2_ mapping population coming from a cross of the dry stem landrace, G21, and a grain sorghum variety, Jiliang 2 whose genome has been re-sequenced. In order to validate the QTL and to narrow down the genomic region harboring the target locus, combined BSA and NGS were employed. The results could help better understand the molecular mechanism underling juice yield of sorghum to improve total sugar yield. Also the introduction of such a dry stalk gene could allow a more timely harvest and avoid additional grain drying expenses.

## Materials and Methods

### Plant materials and method of cultivation

The maternal line for mapping population construction, G21 is a local landrace featured with dry stalk. The stem percent moisture at dough stage (30 to 40 days after flowering) is around 50%. The paternal line is Jiliang 2, an elite sorghum cultivar widely used for sorghum production in North China with stem moisture of around 75% at dough stage. By crossing G21 with Jiliang 2 and selfing the F_1_, 611 F_2_ progenies, together with 30 of each parental line and 20 F_1_ individuals were planted at Shijiazhuang experimental station (38°04′N, 114°29′E) in the year of 2012. The planting density was 40 × 20 cm.

### Phenotypic evaluation

The plants were harvested manually at dough stage (30 to 40 days after flowering) by cutting the plant near the soil surface. The stalk was cut at the nodes after the panicle was excised at the flag leaf and all leaves removed. Fresh stalk weight (FW) of each internode was measured before drying in an oven at 220°C for 48 hrs. Then the stalk dry weight (DW) was measured. The percent moisture was calculated as the following equation:

Percent moisture=(FW-DW)×100/FW.

### SSR marker analysis

DNA was extracted from young leaves using CTAB according to Doyle [25]. To obtain polymorphic SSR markers between G21 and Jiliang 2, SSR markers covering the sorghum genome were first surveyed with the two parental lines. The informative SSR markers identified by this screening were then used for genotyping the F_2_ individuals. The PCR reaction system was composed of 50 ng genomic DNA, 100 ng primer pair, 125 μM dNTPs, 50 mM KCl and 10 mM Tris-HCl, 2 mM MgCl_2_, and 1 unit Taq polymerase. The amplification procedure consisted of one cycle at 94°C for 3 min, followed by 35 cycles of 1 min at 94°C, 1 min at 55 to 58°C depending on the primer pair, 1 min at 72°C, and a final extension step at 72°C for 8 min. The PCR products were separated on a 5% polyacrylamide gel followed by silver staining.

### Whole genome sequencing of bulked DNAs

#### Construction of segregating pools

Two DNA bulks for sequencing were made by selecting extreme individuals from the F_2_ mapping population of 611 plants with the percent moisture ranged from 49–86%. The bulk W (wet) was made by mixing equal amounts of DNA from 50 highly wet stalk plants with percent moisture above 75%, and the bulk D (dry) was made by mixing equal amounts of DNA from 50 highly dry stalk plants with percent moisture below 57%. DNA quality and concentration were measured by 0.8% agarose gel electrophoresis, and adjustments were made for a final DNA concentration of 100 ng/mL.

#### Genomic DNA digestion and amplification

Together with the parental lines, G21 and Jiliang 2, the bulked pools were sequenced on an Illumina GAIIx machine (Illumina, San Diego, CA, USA) according to Biomark’s instruction [26]. Briefly, genomic DNA from the parental lines and both bulked pools were incubated at 37°C with 0.6U *Mse*I (New England Biolabs, Hitchin, Herts, UK), T4 DNA ligase (NEB), ATP (NEB) and *Mse*I adapters. Restriction-ligation reactions were heat-inactivated at 65°C and then digested in an additional restriction with enzymes *Hae*III and *Bfa*I at 37°C. Then PCR was performed containing the diluted restriction-ligation samples, dNTP, Taq DNA polymerase (NEB) and *Mse*I-primer containing a unique barcode for each sample. The PCR products were purified by using E.Z.N.A.H Cycle Pure Kit (Omega) and pooled.

#### Fragment selection, extraction and amplification

The pooled sample was incubated at 37°C with *Mse*I, T4 DNA ligase, ATP and Solexa adapters. The samples were purified using a Quick Spin column (Qiagen) and then run out on a 2% agarose gel to isolate the fragments between 300 to 500 bp in size using a Gel Extraction Kit (Qiagen). These fragments were then subjected to PCR amplification with Phusion Master Mix (NEB) and Solexa amplification primer mix. Phusion PCR settings followed the Illumina sample preparation guide. Samples were gel-purified, and products with appropriate sizes (300 to 500 bp) were excised and diluted for sequencing by Illumina GAIIx (Illumina, San Diego, CA, USA).

#### Sequencing and sequence analysis

The cluster density was optimized to ensure that the specific-locus amplified fragments corresponding with the set requirements, then sequencing of the PCR amplified products was performed on an Illumina GAIIx high-throughput sequencing platform (Illumina, CA, USA). The specific-locus amplified fragments were identified and filtered to ensure that the original sequencing data were effectively obtained. They were clustered based on similarity by employing BLAT [27] and their sequences were obtained through focused recognition and correction techniques.

### Data analysis

#### SSR linkage analysis

The mapping data were analyzed using MAPMAKER/EXP version 3.0b [28], using the Kosambi map function to calculate genetic distances. Linkage was determined at the LOD threshold of 3.0 with a maximum map distance of 50 centiMorgan (cM). The map positions of the markers were visualized using the software Windows QTL IciMapping version 3.2 (http://www.isbreeding.net).

QTL analysis to detect main effect QTL was conducted by using Windows QTL IciMapping version 3.2 following the inclusive composite interval mapping of additive (ICIM-ADD) module within the software. Regions with a LOD score above 3.5 were considered as suggestive of a QTL. Additive QTL was detected using a 1.0 cM step in scanning. The probability used in stepwise regression was 0.001. Threshold LOD scores for detection of definitive QTL were also calculated based on 1000-permutations. Type I error rate to determine the LOD threshold from permutation tests was 0.05 [29].

#### Association analysis

All the obtained markers were identified as to their parental origin of alleles, M (P1) and P (P2), according to the sequencing depth. Maa represents the depth for dry stem phenotype from the maternal line, Paa represents the depth for dry stem phenotype from the paternal line, Mab represents the depth for wet stem phenotype from the maternal line, Pab represents the depth for wet stem phenotype from the paternal line. Ratio_aa = Maa/Paa, when Paa = 0, the Ratio_aa = 1000; Ratio_ab = Pab/Mab, when Mab = 0, Ratio_ab = 1000. Then the ratio of the two groups (aa: dry stem; ab: wet stem) were calculated. The thresholds for association were set at ratio ab> = 3 & ratio_aa> = 1.

### Gene ontology (GO) analysis of selected candidate genes

Candidate genes were submitted to AgriGO (Go analysis tool kit and database for agriculture community) (http://bioinfo.cau.edu.cn/agriGO/index.php) with the sorghum reference genome BTx623 as background. The over represented genes that fell into three categories including biological process, cellular component and molecular function, were filtered by statistical information including Fisher’s exact test and the Bonferroni for multi-test adjustment method [30].

## Results

### Sorghum stem moisture changes

#### Stem moisture changes of Jiliang 2 and G21 in different internodes

**Fig 1:**
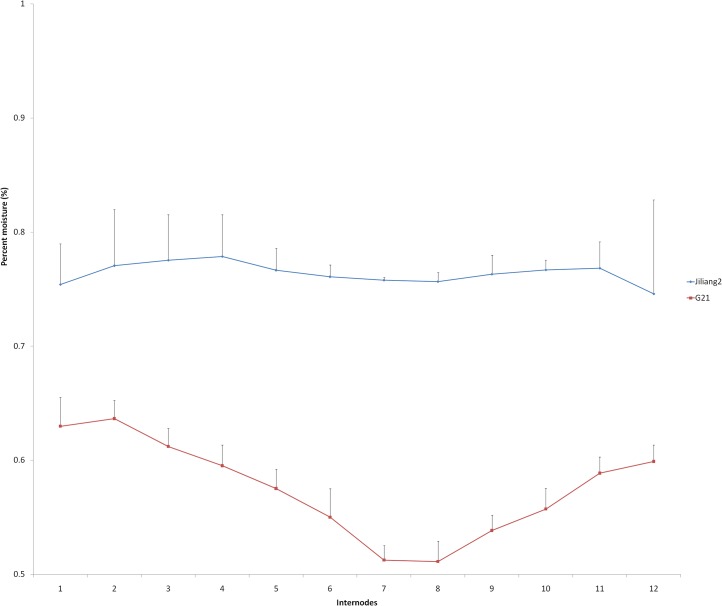
Stem moisture of Jiliang 2 and G21 in different internodes at dough stage. The X axis shows internodes 1–12 whose order was named from the ground surface to spike. The water contents are denoted on the Y axis.

Dough stage (30–40 days after flowering) is the best time for sweet sorghum harvesting. Fig 1 shows the percent moisture of Jiliang 2 and G21 in different internodes at this growing stage. The stalk water content of G21 was much lower than that of Jiliang 2 in all internodes. Further, for Jiliang 2 the water contents in different internodes were relatively the same, the highest is 77.9% of the fourth internode and the lowest is 74.7% of the 12^th^ internode. For G21, the water contents in different internodes were quite different. The 1-3^rd^ internodes have the highest water contents, 63.0, 63.7 and 61.2%, respectively. The lowest water contents were with the internodes in the middle, the 7-8^th^, with 51.3 and 51.1%, respectively (Fig 1).

#### Stem moisture changes of Jiliang 2 and G21 at different growth stages

**Fig 2:**
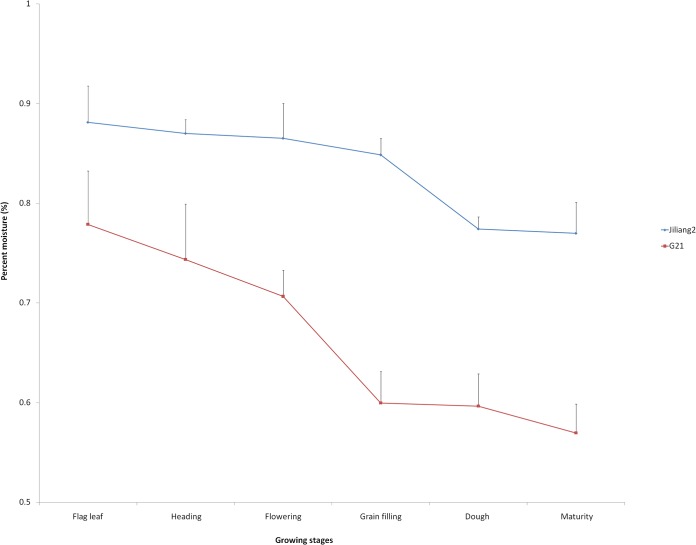
Stem moisture changes of Jiliang 2 and G21 at different growing stages. The growing stages are flag leaf stage (top leaf fully developed), heading stage (spike fully sprouted), flowering stage, grain filling stage, dough stage (kernel well formed and filled with starch) and maturity stage.

The water content of Jiliang 2 was relatively uniform in different growing stages; from flag leaf stage to grain filling stage, the percent moisture of the internode changed little, they were 88.1, 87.0, 86.5 and 84.9%, respectively. During grain filling stage, it dropped to 77.4%, and until maturity stage, 77.0%, it remained stable. However, the changing trend of percent moisture in G21 was quite different from that of Jiliang 2. From flag leaf stage, the water content decreased continuously, especially from flowering to grain filling stage, it dropped from 70.7% to 60.0%. After grain filling stage, the water content was relatively stable until maturity (Fig 2).

### Identification of putative QTL via linkage analysis

**Fig 3:**
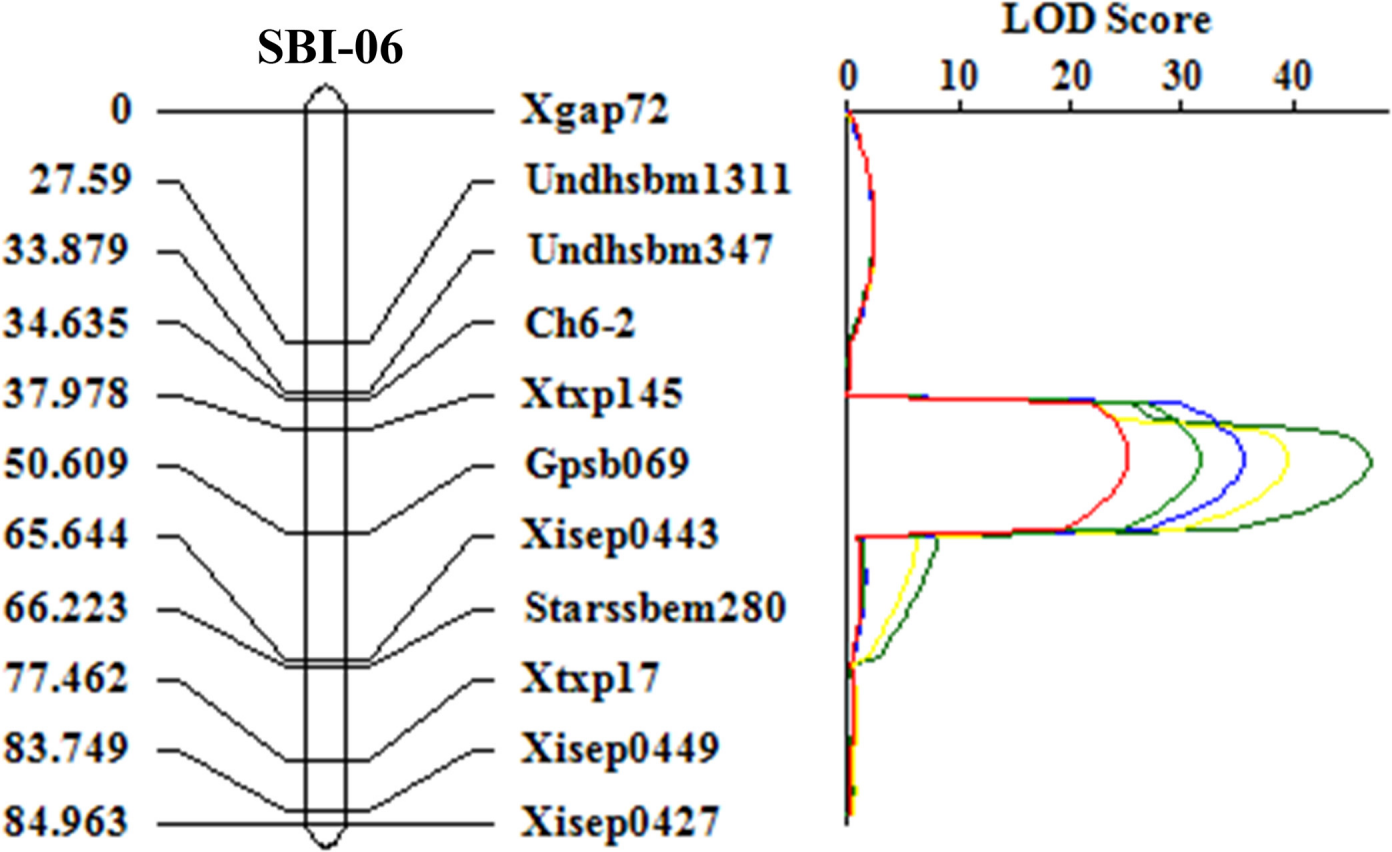
Linkage map of the region harboring *qSW6* on chromosome 6 in the “G21/Jiliang 2” F_2_ mapping population. The genetic distances (cM) between adjacent markers are shown on the left, whereas the names of mapped markers are on the right. The trait was calculated with five different internodes (internode 6–10, in different color), respectively. The LOD scores are indicated on the right of the linkage map. *qSW6* was positioned between markers Ch6-2 and gpsb069.

A total of 326 simple sequence repeat (SSR) markers selected according to their uniform distribution throughout the 10 chromosomes of sorghum were used to initially screen polymorphisms between G21 and Jiliang 2. Among them, 141 markers were polymorphic between the two parents. After screening the F_2_ population with the polymorphic SSR markers, the genotype was analyzed by employing the MAPMAKER program [28]. Linkage was determined at the logarithm of odd (LOD) threshold of 3.0 with a maximum map distance of 50 centiMorgan (cM). The map positions of the markers were visualized and QTL analysis was conducted using the software Windows QTL IciMapping version 3.2 (http://www.isbreeding.net). One major QTL associated with the dry stalk character was identified by using the ICIM mapping program (Table 1). The results show that the 11 markers (S1 Table) covered a genetic distance of 85.0 cM on chromosome 6. The major QTL on chromosome 6, designated as *qSW6* (quantitative trait locus for stem water content on chromosome 6) was mapped between markers Ch6-2 and gpsb069 at 15.0 cM apart (Fig 3) which explained 34.7, 41.6, 45.9, 49.8 and 56.7% of the phenotypic variation with LOD scores of 25.2, 31.5, 35.5, 39.5 and 46.8 with the 5^th^ to 10^th^ internodes, respectively. According to the genomic sequence of chromosome 6 [31], the physical position of Ch6-2 starts from 48,320,691 bp, while Gpsb069 starts from 52,840,234 bp. The two markers covered approximately 4,519 kb.

**Table 1:** The trait name, peak positions (cM), flanking markers, LOD scores, phenotypic variations explained (PVE), additive(ADD) and dominant (Dom) effects and physical genomic position (start-end) of quantitative trait loci (QTLs) detected for water contents using “G21/Jiliang 2” F_2_ population.

TraitName	Position(cM)	LeftMarker	RightMarker	LOD	PVE(%)	Add	Dom	Start-end
stem6	35.0–50.0	Ch6-2	gpsb069	25.2	34.7	-0.0488	-0.0392	48,320,691
stem7	35.0–50.0	Ch6-2	gpsb069	31.5	41.6	-0.0576	-0.0458	52,840,234
stem8	35.0–50.0	Ch6-2	gpsb069	35.5	45.9	-0.0594	-0.0506	
stem9	35.0–50.0	Ch6-2	gpsb069	39.5	49.8	-0.0627	-0.0461	
stem10	35.0–50.0	Ch6-2	gpsb069	46.8	56.7	-0.0655	-0.045	

### Identification of candidate genes via bulked segregant analysis and next generation sequencing

**Fig 4:**
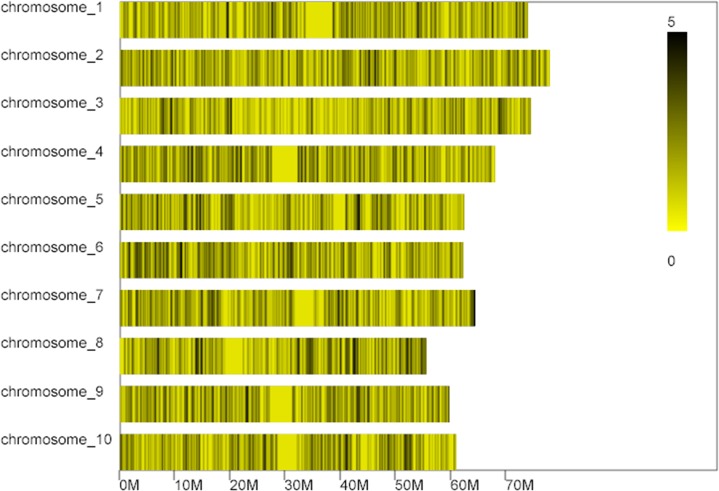
The distribution of markers on each chromosome. The X axis indicates physical position in megabases. The color bar shows the marker density.

Totally 5368 markers mostly (94.45%) single nucleotide polymorphisms (SNPs), some (2.61%) enzyme position single nucleotide polymorphism (EPSNP), and insertion deletion (INDEL) markers (2.91%) were identified through sequencing. Of these, 4983 markers were localized on specific chromosomes (Table 2, Fig 4).

**Table 2:** The distribution of markers on each chromosome.

Chromosome	Marker number
chromosome_1	523
chromosome_2	614
chromosome_3	456
chromosome_4	551
chromosome_5	453
chromosome_6	542
chromosome_7	472
chromosome_8	445
chromosome_9	447
chromosome_10	450
**Total**	4983

**Fig 5:**
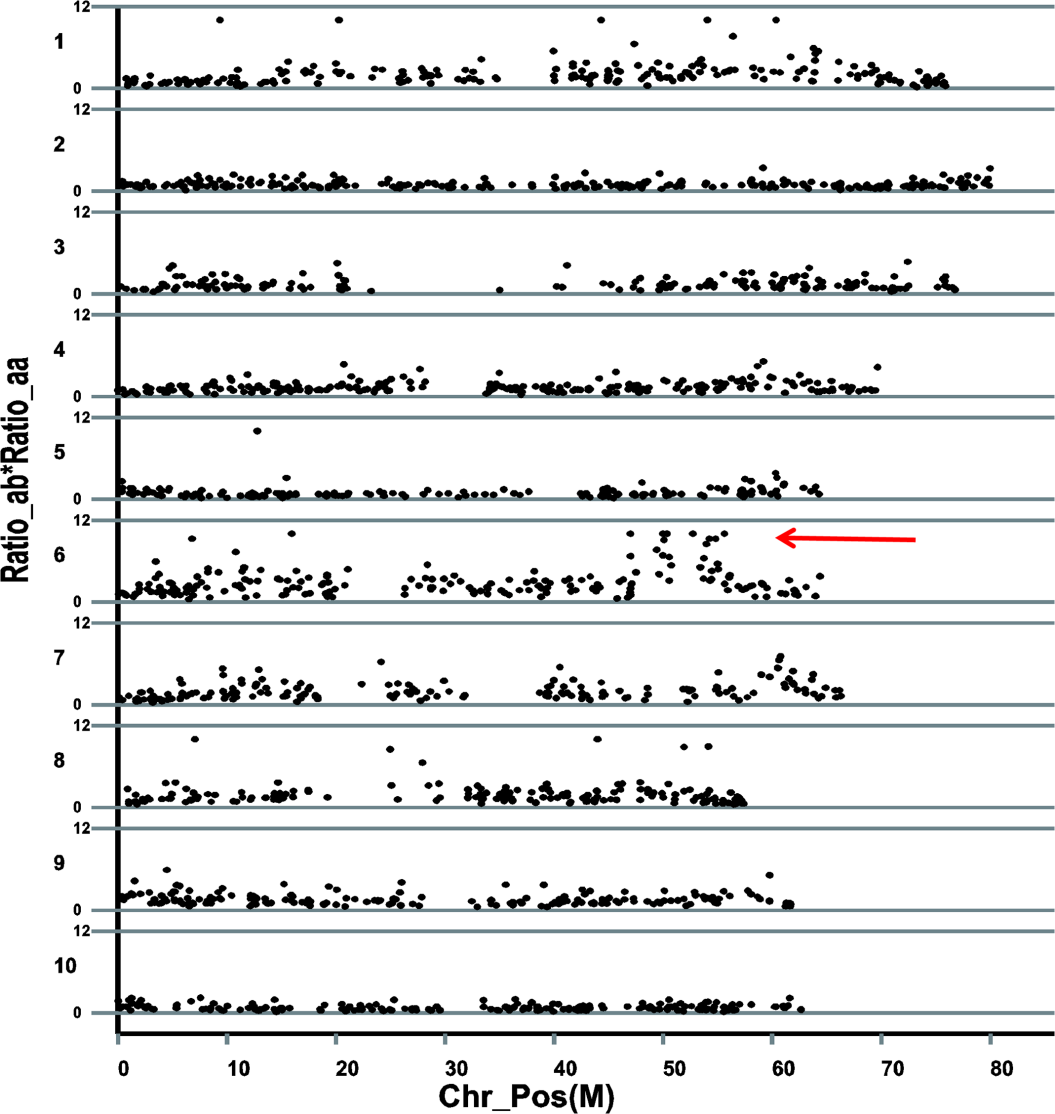
Identification of differentiated markers. The physical positions (in megabases) are denoted on the X axis. The putative associated genomic region is arrowed.

Thirty seven markers (Ratio_ab> = 3 & Ratio_aa> = 1) were identified to be significantly different between the two parental lines and bulked pools on chromosome 1, 3, 4, 6, 7 and 8. Among them, major different markers were located on chromosome 1 (10) and 6 (16) (Table 3). According to the results of difference ratios, the main different markers between the two parental lines and two bulked pools are distributed in a 45–50 Mb region on chromosome 6 (Fig 5). After association analysis, a 339 kb genomic region starting from 48,279,000 to 48,618,000 bp on chromosome 6 with 3 different markers was defined to be associated with the target trait, which contains 38 annotated genes. Comparing with the genomic region of the two flanking markers (48,320,691 to 52,840,234 bp), the candidate region was narrowed down from 4,519,543 to 339,000 bp.

**Table 3:** Chromosome distribution of differentiated markers.

Chromosome	Marker number
chromosome_1	10
chromosome_3	2
chromosome_4	2
chromosome_6	16
chromosome_7	4
chromosome_8	3
**Total**	37

### Candidate gene annotation

**Fig 6:**
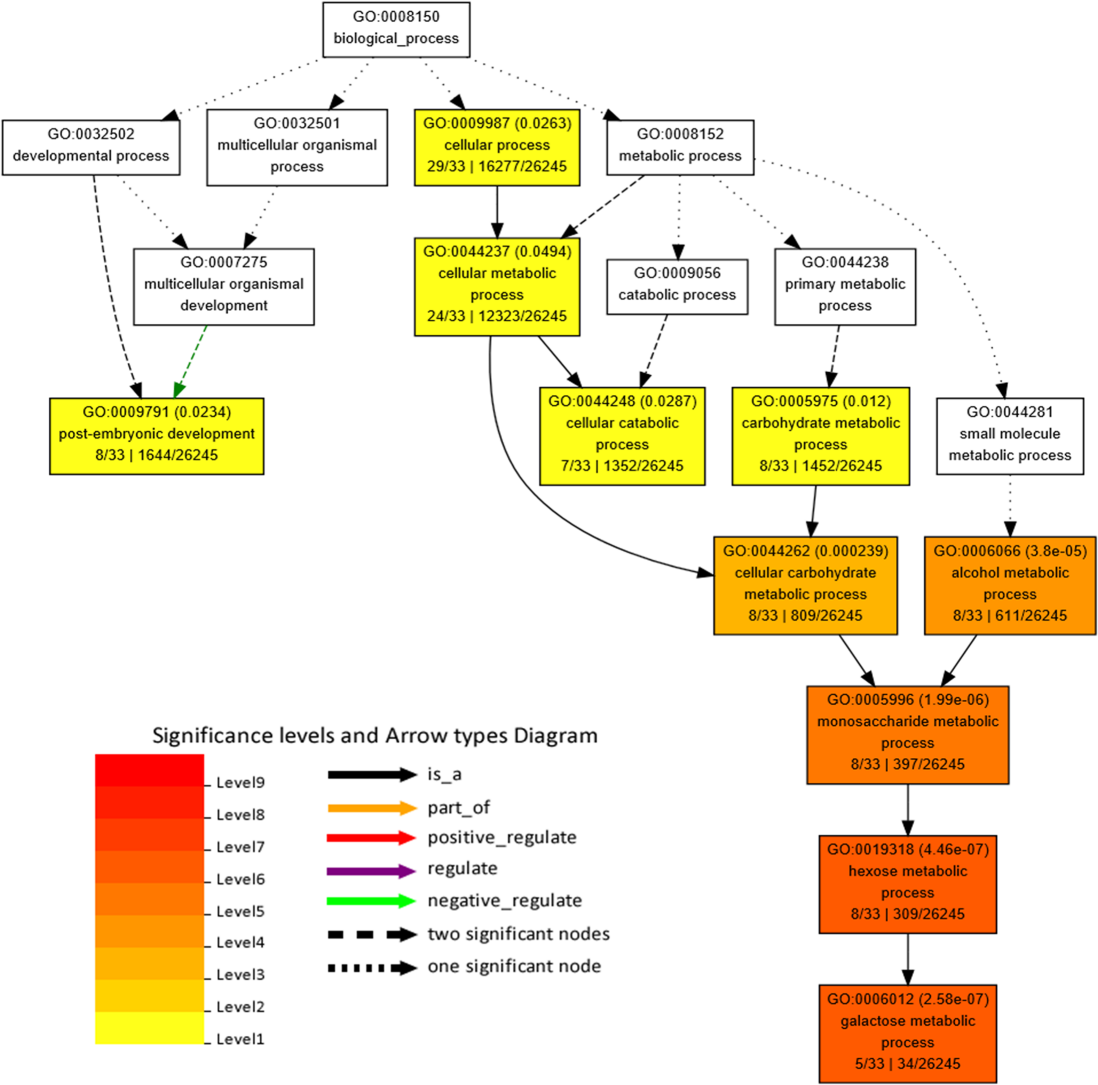
Gene Ontology (GO) analysis of the candidate genes using AgriGO. Each box shows the GO term number, the *p*-value in parenthesis, and GO term. The first pair of numerals represents the number of genes in the input list associated with that GO term and the number of genes in the input list. The second pair of numerals represents the number of genes associated with the particular GO term in the sorghum database and the total number of sorghum genes with GO annotations in the sorghum database. The box colors indicates levels of statistical significance with yellow = 0.05; orange = e-05 and red = e-09.

Within the associated 339 kb genomic region, 38 candidate genes have been identified. GO based functional enrichment analysis of above candidate genes was performed by the web-based tools AgriGO (Go analysis toolkit and database for agriculture community) (http://bioinfo.cau.edu.cn/agriGO/index.php). Singular enrichment analysis (SEA) in AgriGO was used to identify enriched GOs. The results revealed that among the 38 candidate genes, 33 were annotated, of which, 12 GO terms showed significant differences between the candidate genes and all the BTx623 genes pre-computated as background reference, including 10 GO terms involved in biological processes and 2 GO terms involved in the category of molecular function (Table 4, Fig 6). The most enriched terms of biological process ontology were cellular- and cellular metabolic process-related, such as developmental process (GO: 0032502), multicellular organismal process (GO: 0032501), cellular process (GO: 0009987), metabolic process (GO: 0008152), cellular metabolic process (GO: 0044237) and cellular catabolic process (GO: 0044248). For the category of molecular functions, candidate genes were enriched in carbohydrate binding and isomerase activity, including the isomerase activity (GO: 0016835) and carbohydrate binding (GO: 0030246).

**Table 4:** Enriched GO categories of the candidate genes in the target region.

GO term	Ontology	Description	Number in input list	Number in BG/Ref	p-value	FDR
GO:0006012	P	galactose metabolic process	5	34	1.30E-09	2.60E-07
GO:0019318	P	hexose metabolic process	8	309	4.40E-09	4.50E-07
GO:0005996	P	monosaccharide metabolic process	8	397	2.90E-08	0.000002
GO:0006066	P	alcohol metabolic process	8	611	7.50E-07	0.000038
GO:0044262	P	cellular carbohydrate metabolic process	8	809	5.90E-06	0.00024
GO:0005975	P	carbohydrate metabolic process	8	1452	0.00035	0.012
GO:0009791	P	post-embryonic development	8	1644	0.00081	0.023
GO:0009987	P	cellular process	29	16277	0.001	0.026
GO:0044248	P	cellular catabolic process	7	1352	0.0013	0.029
GO:0044237	P	cellular metabolic process	24	12323	0.0024	0.049
GO:0030246	F	carbohydrate binding	5	384	0.00012	0.0037
GO:0016853	F	isomerase activity	5	367	0.000095	0.0037

Abbreviation: P: biological process; C: celluar component; F: molecular function; FDR: false-discovery rate

## Discussion

Traditional gene mapping and map based cloning require identification of markers which are closely flanking and co-segregate with the respective locus [32]. Bulked segregant analysis (BSA), initially developed by Michelmore et al. [33], is an efficient method for the rapid identification of molecular markers linked to any specific gene or genomic region. Any polymorphic marker with clear differentiation of the two bulks will be closely linked to the respective phenotype. However, for candidate gene identification, DNA marker development and genotyping is required. The availability of DNA markers was the main factor limiting effectiveness of the methods. Furthermore, genotyping of each marker for the two bulked DNAs is still time-consuming and costly [24]. Next generation sequencing technologies are being employed to accelerate fine-mapping and gene isolation in many different ways. One of the approaches, combined with bulked segregant analysis, could overcome the limitation of DNA marker availability and avoid complete genotyping. The strategy has been successfully applied for fast gene and/or QTL identification and isolation in wheat [22], rice [24,34], and sunflower [35]. In the present study, a QTL linked with stem water content was initially mapped between two SSR markers at 15 cM apart. By employing next generation sequencing with bulked segregant analysis technique, the linked genomic region was quickly narrowed down to 339 kb. Thus our study proved an efficient strategy to fast and cost-effectively identify quantitative locus responsible for complex trait variation, which could help to understand the underlying molecular mechanism of phenotypic variation and accelerate improvement of crop breeding.

A standard assumption considering moisture content is related to stalk structure type, ie pithy (dry) or juicy. The stalk type can be visually observed and classified. It is usually considered that pithy stem is dryer (lower percent moisture) than juicy stems. One major gene, *d*, determines if a plant has pithy or juicy stems [36]. This gene has been mapped on chromosome 6, linked with a SSR marker Xtxp97. However it was mapped using the midrib as the phenotypic trait observed, not the stem structure appearance [37]. A recent study concluded that, at least in their plant materials, the pithy trait is a major gene that controls the appearance of the midrib and a large portion of the visible stem structure, but it does not influence percent moisture as previously thought. Percent moisture is heritable but is a quantitative trait [18].

Besides the importance of stem moisture as a main factor to determine sugar yield in sweet sorghum, stem moisture also plays an important role in affecting the harvest time of grain sorghum which is very similar with sweet sorghum for most characters. Correct timing of harvest is crucial in order to maximize grain yield, and to minimize grain damage and quality deterioration. Moisture is a key factor to prevent spoilage and avoid the likelihood of additional grain drying expenses. Commercial forage sorghum varieties coming from crosses of *Sorghum bicolor* × *Sorghum Sudanese* with dry stalk character have been released to allow a more timely harvest and help to get the crop bailed and out of the field before it gets rained on while drying (http://www.speareseeds.ca/shared/media/editor/file/Sweet Six BMR Dry Stalk.pdf.). We therefore assume that a drying stem would possibly lead to faster grain drying and lower grain moisture at harvest time, which typically is not a factor in sweet sorghum production. In a previous study regarding relationships of stay green trait in maize, the grain moisture at harvest time was highly significantly correlated with stalk water content [38]. Thus the fine mapping of the dry stalk locus in present study could be applied in marker assisted selection program for more timely harvest in both biomass and grain sorghums and shed some light on other important crops like corn and rice etc. However further study needs to be conducted to prove the assumption.

## Supporting Information

S1 Table Information of the SSR markers linked with the target gene on chromosome 6. (DOCX)
