# Supplementary material for: Combining Next Generation Sequencing with Bulked Segregant Analysis to Fine Map a Stem Moisture Locus in Sorghum (Sorghum bicolor L. Moench)
Source: PLoS One. 2015 May 18;10(5):e0127065. doi: 10.1371/journal.pone.0127065 (PMC4436200; doi:10.1371/journal.pone.0127065)
Supplement: S1 Table — (DOCX) [file pone.0127065.s001.docx]

**Table S1.Information of the SSR markers linked with the target gene on chromosome 6.**

| **Marker name** | **Forward primer sequence (5'-3')** | **Reverse primer sequence (5'-3')** | **Repeat motif** | **Annealing temprature (°C)** |
| --- | --- | --- | --- | --- |
| Xgap72 | TGCCACCACTCTGGAAAAGGCTA | CTGAGGACTGCCCCAAATGTAGG | (AG)_16_ | 55 |
| Undhsbm1311 | GTCCTACGGCAGGTTCTGG | TCAGAGCTCCACCTTCTTGG | (GGCGCT)_2_(GGCGCC)_2_ | 54 |
| Undhsbm347 | ACGCTCTTCACCGTCGAG | TCACACAGGCAGGATCAGAG | (GCGGCA)_2_(GGAGAC)_2_ | 55 |
| Ch6-2 | ACCGCAAGATTTGATGTGAC | TGCTGAAATAGTGCGGAAGA | (GT)_5_(AT)_18_ | 54 |
| Xtxp145 | GTTCCTCCTGCCATTACT | CTTCCGCACATCCAC | (AG)_22_ | 55 |
| Gpsb069 | CCCATAATACTTGACCTTC | ACTTACTCCCTCTGTCCC | (TC)_12_ | 55 |
| Xisep0443 | TCATGTACAGAGGCGACACG | AGGTCGCAACAGACACCTTC | (GCA)_7_ | 55 |
| Starssbem280 | ACCAACCTGCCTACCATCAG | GAGCGAGAGGCTGAGGACT | (GCTC)_5_ | 55 |
| Xtxp17 | CGGACCAACGACGATTATC | ACTCGTCTCACTGCAATACTG | (TC)_16_+(AG)_12_ | 55 |
| Xisep0449 | CCGCTCATCAGTCATCACAT | ACAAAATCCATCCCACAACG | (TCA)_7_ | 55 |
| Xisep0427 | AAGCGGCGGAAAGAGAAG | GAGCGAGAGGCTGAGGACT | (GA)_6_ | 55 |
